# Association between pre- and post-workout protein intake and mid-arm muscle circumference in adolescent swimmers

**DOI:** 10.1080/15502783.2025.2550203

**Published:** 2025-09-17

**Authors:** Larissa de Brito Medeiros, Maria Rayssa Silva do Nascimento, Hellenrita Lima da Luz Castro, José Atson da Silva Barbosa, Rafael Ikaro de Souza Carvalho

**Affiliations:** João Pessoa University Center – UNIPÊ, Nutrition Program, João Pessoa - PB, Brazil

**Keywords:** Dietary intake, protein timing, muscle mass, performance

## Abstract

**Background:**

This study aimed to evaluate pre- and post-workout protein intake and its association with mid-arm muscle circumference (MAMC) in adolescent swimmers.

**Methods:**

A cross-sectional study was conducted with 22 adolescent swimmers (both sexes) from a sports center in Paraíba, Brazil. Protein intake before and after training was assessed using 24-hour dietary recalls and compared to the recommendations of the International Society of Sports Nutrition (ISSN). MAMC was calculated and classified according to the 50th percentile for age and sex, based on reference values from the National Health and Nutrition Examination Survey (NHANES). Statistical analysis was performed using IBM® SPSS® Statistics version 20.0. Associations between categorical variables were tested using the Chi-square test, with significance set at *p* < 0.05.

**Results:**

Although no statistically significant association was found between protein intake (pre- or post-workout) and MAMC (*p* = 0.530), the majority of participants reported protein intake below recommended levels in the post-workout period (59%, *n* = 13) and above recommended levels in the pre-workout period (91%, *n* = 20). While pre- and post-training protein intake may not fully represent total daily protein consumption, the timing of intake relative to physical activity plays a key role in muscle recovery and, consequently, athletic performance.

**Conclusions:**

These findings underscore the importance of not only meeting daily protein requirements but also optimizing protein distribution and timing. Although no significant association was found between protein intake and MAMC in this sample, the study highlights the relevance of nutritional strategies that align protein intake with exercise timing to support muscle development and performance

